# A narrative review on the non-surgical treatment of chronic postoperative inguinal pain: a challenge for both surgeon and anaesthesiologist

**DOI:** 10.1007/s10029-022-02693-9

**Published:** 2022-10-31

**Authors:** N. van Veenendaal, N. B. Foss, M. Miserez, M. Pawlak, W. A. R. Zwaans, E. K. Aasvang

**Affiliations:** 1 Department of Anesthesiology, University Medical Center Groningen, Hanzeplein 1, 9713 GZ Groningen, The Netherlands; 2 Department of Anaesthesia and Intensive Care, Hvidovre University Hospital, Copenhagen, Denmark; 3 Department of Abdominal Surgery, University Hospitals Leuven, Leuven, Belgium; 4 North Devon Comprehensive Hernia Centre, North Devon District Hospital, Royal Devon University Healthcare NHS Foundation Trust, Barnstaple, UK; 5 Department of General Surgery, Máxima Medical Center, Veldhoven, Eindhoven, The Netherlands; 6 SolviMáx Center of Excellence for Abdominal Wall and Groin Pain, Eindhoven, The Netherlands; 7 NUTRIM School of Nutrition and Translational Research in Metabolism, Maastricht University Medical Center, Maastricht, The Netherlands; 8 Department of Anesthesiology, Center for Cancer and Organ Diseases, Rigshopitalet, Copenhagen, Denmark; 9 Department of Clinical Medicine, Copenhagen University, Copenhagen, Denmark

**Keywords:** Chronic pain, Inguinal hernia repair, Groin hernia repair, Chronic postoperative inguinal pain

## Abstract

**Introduction:**

Chronic pain is one of the most frequent clinical problems after inguinal hernia surgery. Despite more than two decades of research and numerous publications, no evidence exists to allow for chronic postoperative inguinal pain (CPIP) specific treatment algorithms.

**Methods:**

This narrative review presents the current knowledge of the non-surgical management of CPIP and makes suggestions for daily practice.

**Results:**

There is a paucity for high-level evidence of non-surgical options for CPIP. Different treatment options and algorithms have been published for chronic pain patients in the last decades.

**Discussion and conclusion:**

It is suggested that non-surgical treatment is introduced in the management of all CPIP patients. The overall approach to interventions should be pragmatic, tiered and multi-interventional, starting with least invasive and only moving to more invasive procedures upon lack of effect. Evaluation should be multidisciplinary and should take place in specialized centres. We strongly suggest to follow general guidelines for treatment of persistent pain and to build a database allowing for establishing CPIP specific evidence for optimal analgesic treatments.

## Introduction

Within the past decades, chronic postoperative pain has gone from sporadically reported, to being recognized as a common and complex problem after all types of surgery. Whereas the overall incidence ranges from 5 to 20% depending on the specific procedure, pain affecting everyday activities occurs in about 5–8% of patients across procedures [1–3]. The Global Burden of Disease Study 2016 showed a 15.3% increase in burden of disease due to abdominal wall hernias [4]. The majority of these hernias require repair, resulting in 20 million groin hernia repairs being performed annually [5, 6].

Chronic postoperative inguinal pain (CPIP) is defined as pain for at least 3 months, including a level of pain rated by the patient as at least moderate and impacting daily activities [7]. Since groin hernia repair has a 10–12% risk for CPIP, this means that two million people are at risk of sustained pain more than three months after groin hernia repair yearly, resulting in a significant global burden of disease [7–9]. Due to the low incidence of hernia recurrences, prevention of CPIP should be at least as high a priority for the hernia surgeon.

The optimal solution for CPIP would be prevention. However, despite several intra-operative strategies (e.g. laparoscopic technique, careful tissue handling, mesh selection, anaesthesiological and analgesic techniques, etc.), it is still impossible to avoid CPIP from occurring in specific patients. This is partially due to inpatient factors, such as patient’s genetics and nociceptive systems, making them susceptible to chronic pain. Thus, we as clinicians are left with the task of managing CPIP, which is difficult due to its complexity and heterogeneity, and the lack of clear evidence based guidelines.

Several approaches have been suggested for the management of CPIP, ranging from cognitive therapies or pharmacological interventions to re-operations with neurectomy and/or mesh removal. Treating CPIP with another surgical intervention sounds contradictory on itself, as surgery for pain could potentially aggravate pain. The essence is “do no further harm” or “doing less is best”. However, reoperation can produce significant improvements and is undoubtedly an effective treatment modality in selected patients [10, 11].

The management of patients with CPIP is complex, and we have to acknowledge that the evidence is still too sparse to allow firm recommendations for daily practice. Currently, studies on surgical interventions for CPIP have inadequate descriptions of preoperative triage processes to make firm conclusions on the actual risk/benefit profile relative to less invasive measures. Nevertheless, promising results are emerging and evidence from other areas within chronic pain can be drawn upon when treating these patients. The aim of this review was to present the current knowledge of the non-surgical management of CPIP, in the context of social, psychological and physical factors of the individual patient. Additionally, suggestions are made for the management of patients with CPIP and future research.

## Methods

For this narrative review we were informed by studies that describe the non-surgical management of CPIP. We searched seven electronic databases (PubMed, PudMed Central, MEDLINE, Embase, the Cochrane Central Register of Controlled Trials, Google Scholar and Springer Link). The following search terms were used: “inguinal hernia”, “groin hernia”, “hernia repair”, “mesh repair”, “CPIP”, “pain”, “inguinodynia”, “non-surgical treatment”, “capsaicin”, “lidocaine”, “pharmacological”, “radiofrequency”, “cryoablation”, “peripheral nerve stimulation”, “DRG”, “centralization”, “expertise”. All titles and abstracts were screened for eligibility by two authors (NVV, EA). In case of disagreement the other authors were consulted. Based on the literature and on personal experience suggestions were made.

## Results

### CPIP definition

Understanding the problem of CPIP renders a clear definition first. Throughout literature the definition of chronic postsurgical pain differs. The original definition of postsurgical pain by Macrae stipulates that the pain has developed after a surgical procedure. Furthermore, the definition includes at least two months duration, and that other causes for the pain as well as other pre-existing problems must have been excluded or solved [12]. According to the International Association for the Study of Pain (IASP), chronic postsurgical pain would be defined as “chronic pain that develops or increases in intensity after a surgical procedure or a tissue injury and persists beyond the healing process, i.e. at least 3 months” [13]. Although time thresholds are included in both definitions, caution should be taken when translating this definition for inguinal hernia surgery.

A time threshold of three months has been suggested by IASP because it provides for clear operationalization [14]. However, the inflammatory healing process in mesh-based inguinal hernia repairs may last longer [15]. A longer time frame of six months was suggested in the definition of CPIP in 2013 [16]. Additionally, it was stated that the postoperative pain should be different from the pre-operative pain in terms of frequency, intensity, location, and character. This is important, as it allows for the presence of pre-operative pain in CPIP, in contrast to the Macrae definition, recognizing that pre-existing pain itself is one of the most important risk factors for developing CPIP and other chronic postoperative pain syndromes [2, 3]. The two definitions are compared in Table 1.

**Table 1:** Definitions of post-surgical pain and chronic postoperative inguinal pain

Chronic post-surgical pain	Chronic postoperative inguinal pain
1. The pain developed after a surgical procedure	1. Pain occurs after a herniotomy
2. The pain is of at least 2 months duration	2. Pain of at least 6 months duration
3. Other causes for pain should have been excluded (e.g. continuing malignancy or chronic infection)	3. Other causes of pain excluded
4. The possibility that the pain is continuing from a pre-existing problem has been explored, and exclusion attempted	4. Postoperative pain different from pre-operative pain (frequency, intensity, location, character)

Despite the various definitions of chronic pain, the Hernia Surge guideline recommends using the widely accepted time period of at least three months to define CPIP, and we agree with that [7]. Additionally, it is recommended that the CPIP definition includes a level of discomfort rated by the patient as at least moderate and impacting daily activities. The instigation of treatment should assess to which extent the pain impacts the patient’s life, to discuss the pro and con’s when deciding whether and how to treat the patient.

### CPIP: What do we already know?

Guidelines have gained much popularity in the last decades, by summarizing the best available evidence and providing recommendations for physicians. They will hopefully become more specific for CPIP in the future, when more evidence is gained, but as for now they are mainly overviews of topics that recommend more research.

Table 2 presents the evidence of the non-surgical treatment of CPIP. In 2018, the first international guideline on groin hernia management was published, including a thorough summary of the latest evidence regarding CPIP until January 2015 [7]. Until then, seven reviews had been published describing different treatment options and algorithms for chronic pain patients [8, 10, 17–21]. Neither any of these reviews or algorithms have been tested for the impact on patient outcomes in large series, nor have found that the evidence for CPIP treatment was solid enough to suggest changes to daily practice.

**Table 2:** Evidence table of non-surgical management options of CPIP

Intervention type	Author	Year	*N* =	Study design	Conclusion	Comment
Pharmacological topical therapy						
Lidocaine patch (5%)	Bischoff [21]	2013	21	Randomized, double-blind, placebo-controlled, crossover trial: lidocaine patch (5%) versus placebo patch	No difference in summed pain intensity differences between lidocaine and placebo patch treatments in all patients	Small-volume study
Capsaicin patch	Bischoff [22]	2014	46	Randomized, double-blind, placebo-controlled, crossover trial: capsaicin 8% patch versus placebo patch	No statistically significant benefit of the capsaicin patch, although there was a trend toward less pain in the capsaicin group at one month	Small-volume study. Only one month follow-up
Pharmacological systemic therapy						
Paracetamol, NSAID and gabapentinoid	No evidence					
Interventions						
Nerve blocks and trigger point infiltrations						
Ultrasound-guided nerve block	Voorbrood [23]	2015	28	Prospective study	Permanent pain reduction was achieved in 18 of 28 (62%) patients with neuropathic pain	
Ultrasound-guided nerve block	Bischoff [24]	2012	12	Randomized, double-blind, placebo-controlled, crossover trial: lidocaine versus placebo	1 lidocaine responder, 6 non-responders, and 5 placebo responders. Ultrasound-guided ilioinguinal nerve and iliohypogastric nerve blocks did not produce pain relief	It is not clear from the study what percentage of patients had improperly-placed nerve blocks despite ultrasound guidance
Ultrasound-guided or nerve stimulator-guided nerve block	Thomassen [25]	2013	43	Retrospective study	Thirty-two percent of the patients were relieved of moderate-to-severe pain and nerve blocks 21 patients (55.3%) no longer reported neuropathic pain Ilioinguinal/iliohypogastric nerve blocks can be effective to treat chronic inguinal pain following surgery of the groin	Nerve stimulator-guided blocks were performed prior to January 2009, and thereafter, ultrasound-guided blocks
Ultrasound-guided or landmark-based nerve block	Trainor [26]	2015	36	Retrospective study	14 patients (70%) in the landmark-based and 11 patients (79%) in the ultrasound-guided groups experienced at least a 50% reduction in VAS scores (*p* = 1.0)	Small-volume study. No information on follow-up
Ultrasound-guided tender point blockade	Wijaya-Singhe [29]	2016	14	Randomized, double-blind, placebo-controlled, crossover trial: bupivacaine 0.25% versus placebo	Median pain reduction of 63% (44.1 to 73.6%) after bupivacaine compared with 36% (11.6 to 49.7%; *p* = 0.003) after placebo	Small-volume study. Short follow-up of 14 days. No difference in movement related pain, summed pain intensity scores, or sleep quality scores
Tender point infiltration (TPI)	Verhagen [10]	2018	54	Randomized controlled trial: tender point infiltration versus neurectomy	TPI was successful in 6 patients (22%), a neurectomy was successful in 17 patients (71%). After unsuccessful TPI, 19 patients crossed over to neurectomy and their median VAS score dropped from 60 to 14 (*p* = 0.001). A step-up treatment strategy starting with tender point infiltration followed by a tailored neurectomy is advised	Although neurectomy seems superior in this trial, minimally invasive techniques are preferred in a step-based approach
CT-guided nerve block	Parris [27]	2010	1	Case-report	CT-guided transpsoas genitofemoral nerve block is a viable option for safely and selectively blocking the genitofemoral nerve for diagnostic or therapeutic purposes	Case-report
CT-guided peri-neural injections	Poh [28]	2019	58	Retrospective study	Improvement was seen in 84% of the cases	Non-controlled, non-randomized study design and unclear duration of effect
Neuroablative techniques and stimulation techniques						
Cryoablation	Fanelli [30]	2003	10	Case series	77.5% mean overall pain reduction, average follow-up period of eight months	Small-volume study. Short follow-up
Peripheral nerve stimulation	Shaw [31]	2016	6	Retrospective study	Average improvement of 62% in the immediate post-operative follow-up. Eighty-five percent patients were completely satisfied with an average follow-up of 22 months	Small-volume study. Non-controlled, non-randomized study design
US-guided microwave ablation	Lee [32]	2019	10	Retrospective study	Immediate pain reduction in 92% of the subjects, and 69% pain reduction at 12 months follow-up. The average duration of clinically significant pain reduction was 10.5 months	Small-volume study. Non-controlled, non-randomized study design
Dorsal Root Gang-lion Stimulation	Schu [33]	2014	12	Case series	Mean VAS reduction of 76.8% ± 8.2%, pain relief in 10 (83%) patients, follow-up period of 17.4 ± 5.7 weeks	Small-volume study with various etiologies of groin pain. Short follow-up
Spinal Cord Stimulation	Yakovlev [34]	2010	15	Case series	Pain relief of > 75% and reduced pain medication intake, follow-up period of 12 months	Small-volume study
Other therapies						
Physical therapy, psychotherapy, hypnosis, behavioural therapy, biofeedback, acupuncture, mind–body therapy	No evidence					

Although limited evidence exists for systemic pharmacological treatments (e.g. acetaminophen/paracetamol, NSAIDs, TCAs, SSRIs, gabapentin, pregabalin, and opioids), a step-wise multidisciplinary approach starting with minimally invasive measures like systemic analgesics is advocated in the seven reviews and the Hernia Surge guideline [7]. Although low invasive, systemic opioids and other centrally acting drugs have significant side effects, and their effect and safety as a first line therapy, relative to minimally invasive interventions such as nerve blocks, are poorly described in studies.

Based on two small studies, lidocaine and capsaicin patches have not been proven to be effective in CPIP patients [22, 23].

Nerve blocks are another option in the management of CPIP that are advocated in all reviews, algorithms and the Hernia Surge guideline [7]. Although the evidence is scarce, nerve blocks are considered to be useful in the diagnostic and therapeutic management of CPIP [11, 24–30]. No evidence-based recommendations for preferred technique (ultrasound-guided, neuro-stimulator directed, anatomic landmark-guided) can be made based on the evidence available and it is left to the discretion of the individual physician (preferably a hernia expert since the optimal site of the block will depend on the surgical approach and anatomical mesh location). However, it is recommended to perform ultrasound-guided nerve blocks in order to obtain optimal visualization of the injection site. Similar to other interventions in CPIP, the studies on nerve blocks have poor descriptions of previous or failed interventional and medical therapies in the included patients.

In addition to the conventional nerve blocks, there is evidence on the diagnostic value of trigger point infiltrations [30]. These local injections are minimally invasive, safe and easy to perform. Therefore, trigger point infiltrations might be an appropriate modality in the early management of CPIP. In future studies trigger point infiltrations should be clearly differentiated from peripheral nerve blocks. Their therapeutic effect as first line therapy should be further studied.

All studies on more invasive conservative treatment modalities for CPIP (e.g. ablation techniques and neuromodulation) have significant limitations, such as small-volume studies, non-controlled and non-randomized designs, short follow-up periods and no report of adverse events or complications [31–35]. In addition, the selection of patients for these studies regarding previous therapeutic interventions is not consistent, precluding conclusions regarding its place in the therapeutic cascade. The initial positive results of these studies should, therefore, be interpreted with caution. Although very low evidence, early findings suggest that pulsed radio frequency ablation and neuromodulation of the Dorsal Root Ganglia (DRG) may be an effective treatment for chronic neuropathic pain conditions in the groin region [7]. Finally, there is no evidence for non-pharmacological and non-surgical interventions such as physiotherapy, psychotherapy, hypnosis, behavioural therapy, biofeedback, acupuncture or mind–body therapy [7].

In the Hernia Surge guideline it was already stated that there is a paucity of non-surgical options for CPIP. Despite many research questions that were raised, relatively few new insights were published in past decade. In future studies the role of trigger point infiltrations needs to be studied and clearly differentiated from peripheral nerve blocks. Additionally, it is suggested that the repetitive effect of more proximal nerve blocks in managing CPIP is explored. Furthermore, the role of central sensitization needs to be studied.

## Discussion

### Current practical suggestions for non-surgical handling of the patient with CPIP

Due to the lack of established evidence-based treatment options for CPIP, we suggest to apply the following strategy for handling CPIP:

Any hernia center should have a formulated policy for how to deal with the inevitable occurrence of CPIP. These include preoperative information of the patient on the risk, assessment of CPIP during postoperative follow-up including use of analgesics (opioids and gabapentinoids in particular) and established collaboration and referral to expert pain centres ideally with an interest in CPIP and/or other chronic postoperative pain syndromes.

**Fig. 1:**
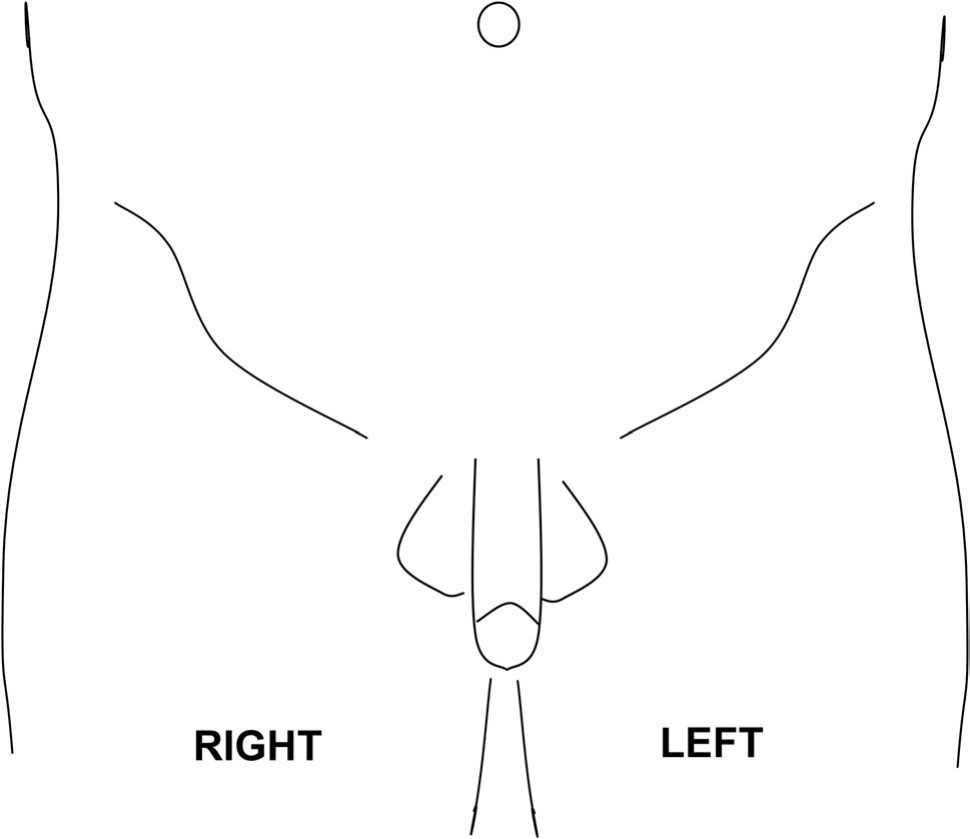
Suggested body map for recording of quantitative sensory testing findings

**Fig. 2:**
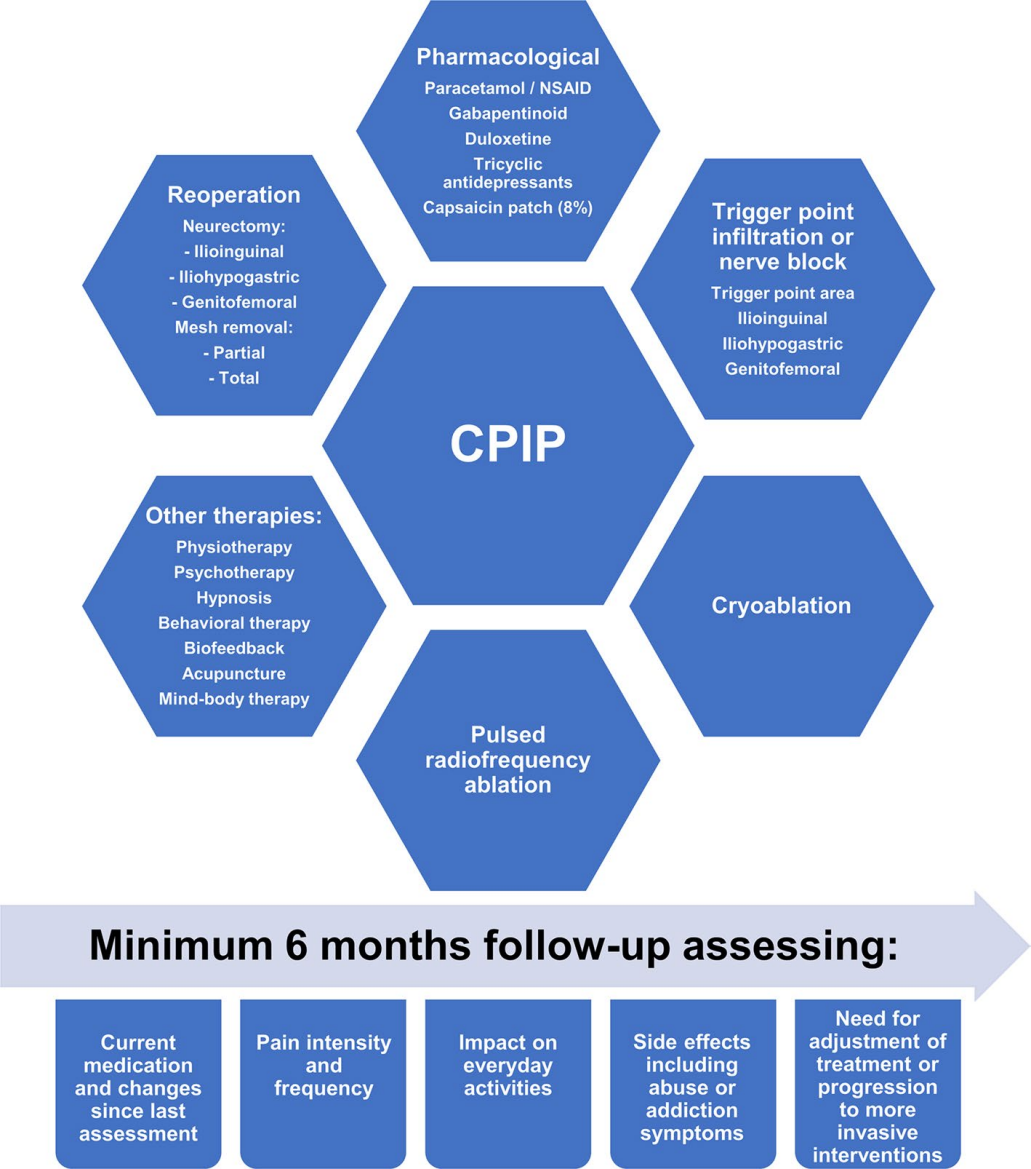
Overview of potential CPIP treatment modalities, assessment, and follow-up

1. **Information:** The patient assessed for a first-time hernia repair should be advised that there is a about 10–12% risk of persistent pain at three months follow-up and a 0.5–6% risk of pain affecting everyday life at one year follow-up, including sexual function [7]. The discussion should be nuanced with the evidence that for the pain-free hernia there is the option of watchful waiting [36]. The patient assessed for a re-operation for CPIP should be informed that although the evidence suggests a positive outcome in a proportion of patients, it is difficult to predict who will benefit and there is a risk for pain intensification [37, 38]. The patient should be advised on non-surgical treatment options (see below).
2. **Assessment:** As for now we suggest that patients with CPIP are referred to dedicated hernia expert centres that should collect core data in a standardized manner to allow for combining these later on with other centres [39]. This will also allow for updated knowledge on potential effective treatments to be offered for the patient, preferably as part of prospective studies. Thus, in line with the earlier suggestion we recommend that the following data are collected as a first step to assess the likelihood of CPIP, monitor treatment efficacy of the individual patient, and collect data for scientific progress overall.
  *History:* As with any chronic pain patient the first approach should be to gather fulfilling information on the surgical and pain history of the particular patient, including previous treatment attempts and results. The intensity, frequency and impact of pain on everyday activities and sexual life should be registered, using validated questionnaires such as the activity assessment scale [40]. Additionally, the psychosocial history and risk factors of patients should be addressed, to identify those at increased risk of developing chronic pain [41].
  *Physical examination:* A physical examination should be performed, describing the painful area including the surgical scar, surrounding skin, genitalia for testicular affection in particular, contralateral side, other potential differential causes (spinal disc herniation, hip arthrosis, hernia recurrence, etc.). These may include radiological investigations, and effect from diagnostic nerve blocks or trigger point infiltrations [30].
  *Sensory examination:* Assessing the occurrence of sensory disturbances such as hypoesthesia, hyperalgesia, allodynia, etc., should be performed in a standardized but everyday clinical feasible way. The precise methodology is not agreed upon, but a relatively simple bedside testing protocol has been suggested with good agreement with the far more elaborate and time consuming Quantitative Sensory Testing methodology (QST), i.e. with cotton swaps, finger pressure, etc. [42]. The suggested test takes less than 5 minutes, and as previously suggested, we recommend that the findings are marked on a standardized body map (Fig. 1). We recommend sensory testing acknowledging that painful and painless neuropathies exist in CPIP [43], as in other conditions [44], but recognizing the emerging evidence that sensory profiling may potentially allow for identifying initial pharmacological treatment with a higher rate of success depending on the individual patients’ characteristics of loss or gain of sensory function [45].

3. **Treatment:** Based on all literature it is clear that we have not yet formed solid evidence to give specific recommendations on the treatment of CPIP patients [7]. Despite the promising results of the surgical interventions for CPIP, we agree that caution should be taken to minimize the risk of pain aggravation by remedial surgery [7]. On the other hand, we must also stress that long-term analgesic treatment, especially opioids and gabapentinoids, have a high risk of adverse effects, including transition into abuse with well-documented increased mortality [46, 47]. Thus, we recommend the overall principle of starting out with the least invasive strategy and advancing into surgical procedures when conservative treatments are to no avail (Fig. 2). Ideally, a future joint effort may identify patients who are eligible for safe and effective surgical interventions early on, and those with a high risk for unsuccessful surgery who should be diverted into other treatments. For now, we recommend establishing a dedicated pain clinic at the hernia institution or collaborating with such to allow referral of patients. We recommend that the overall approach to interventions should be pragmatic, tiered and multi-interventional, starting with least invasive and only moving to more invasive treatments upon lack of effect. This will have the potential to minimize high-intervention procedures and potentially be more cost-effective with fewer side effects. Treatment of patients should start out by following the overall guidelines for persistent (neuropathic) pain treatment, including gabapentine, duloxetine and tricyclic antidepressants [48, 49]. However, as with all pain conditions there is an inflammatory component in CPIP and the benefit of paracetamol in combination with NSAIDs or COX-II inhibitors should not be excluded. This also implies attention to the well-known caveats in case of renal or cardiac failure and especially the risk of GI bleeding from long-term treatment, which can be prevented by proton-pump inhibitors. The initial step can also include treatment with capsaicin patches which in contrast to local-analgesic patches have shown effect on localized neuralgias [34] and is of particular interest due to the non-systemic effect. Second line treatments include tramadol due to the serotonine-noradrenaline reuptake inhibitory effect, whereas pure opioid agonists (i.e. morphine and oxycodone) are not recommended due to the moderate effect in (neuropathic) CPIP and high risk for adverse effects, including abuse.
  Third line treatments include (repeated) nerve blocks and trigger point infiltrations, whereas cryoablation and pulsed radiofrequency are still considered with low evidence for efficacy requesting further evidence. For patients without satisfactory effect, we believe that the possibility of re-operation with (partial) mesh removal and/or neurectomy can be discussed, but lies outside the scope of this review.
4. **Evaluation and follow-up:** Regardless of the specific treatment chosen in the various steps described above, we consider it pivotal that follow-up should be standardized regarding assessment and time, with defined success criteria for continuation of treatment or initiation of other options. These also include assessing the side-effects from pharmacological treatments such as dizziness, cognitive impairment and potential abuse.

### Future perspectives

Since there is a paucity of high-level evidence on best practices, more evidence needs to be created. Additionally, low-level evidence needs validation.

One of the major challenges in the data on both non-surgical and surgical interventions is the selection of patients in the individual trials. The scarce descriptions of any actual effect of previous interventions, as well as the lack of potential effects of less invasive interventions in the included patients, limits the ability to place the individual therapies in a rational staggered approach. This can only be solved by creating well-defined inclusion data and follow-up between interventions, ideally even standardizing the order of interventions.

Due to the scarcity of evidence, we suggest that a database including all treated patients is created. This can be a database on the local, national, or international level. Ideally a centralized database is created with core data collected at all sites, allowing merging of information and multicentre studies for large trials or in cases of rare patient findings. Such a database structure should also allow for individual centres to add local investigations and treatment strategies but informing about this as to make sure data are comparable. The database should not apply to research projects only, but collect data from all treatments being performed at the participating centres. It is crucial that all CPIP patients treated in these centres are included in this database to avoid selection bias. We suggest that the hernia-surgery community formulates an assessment algorithm with standardized treatment suggestions similar to the approach described above. This will allow for large data on specific treatment strategies regarding efficacy and side effects, as we believe the time has come to move away from the predominantly small single-centre studies. Besides describing the effect of the individual interventions, such a database will also allow the relative effects to be assessed, facilitating the establishment of an evidence-based treatment order of invasive interventions, optimizing benefit and minimizing harm. Formulating such an algorithm will undoubtedly be an advantage, also to local centres not participating, due to the constantly updated evidence-based best-practice being formulated.

Last, we suggest that the optimal management of CPIP patients should be centralized in regional, specialized hernia centres. Since the volume of hernia surgery in expert hernia centers reports lower incidences of CPIP, the treatment of CPIP patients should ideally be centralized as well. The management of CPIP has been proven to be very challenging, requiring a multidisciplinary team approach with dedicated professionals. Establishment of such expert centres forms the basis for creating evidence-based treatment algorithms for CPIP patients and initiating the databases mentioned before. We believe this is pivotal for the success of CPIP treatment in the future.

### Limitations

It is difficult to draw conclusions on the management of CPIP given the scarcity of high-level evidence in this field. Literature has various definitions of CPIP; studies show heterogeneity of study populations and study cohorts are relatively small. This impacts the generalizability of results, limiting the applicability of study results in new CPIP patients.

## Conclusion

CPIP is one of the most frequent clinical problems after inguinal hernia surgery and despite more than two decades of research and numerous publications, no evidence exists to allow for CPIP specific treatment algorithms. We suggest that non-surgical treatment is introduced in the management of all CPIP patients. The overall approach to interventions should be pragmatic, tiered and multi-interventional, starting with least invasive and only moving to more invasive upon lack of effect. Evaluation should be multidisciplinary and should take place in specialized centres. We strongly suggest to follow general guidelines for treatment of persistent pain and to build a database allowing for establishing CPIP specific evidence for optimal analgesic treatments.
